# Peripheral nerve injury associated with JEV infection in high endemic regions, 2016–2020: a multicenter retrospective study in China

**DOI:** 10.1080/22221751.2024.2337677

**Published:** 2024-04-05

**Authors:** Guowei Wang, Lianmei Zhong, Manxia Wang, Juan Zhou, Shuting Liu, Wang Miao, Leilei Li, Yonghong Liu, Shougang Guo, Haining Li, Xiaoming Wang, Liuqing Xie, Min Xie, Shihong Fu, Tingting Xuan, Fan Li, Tingting Yang, Lufei Shao, Mingfang Shi, Xiaocong Li, Xiaoling Li, Li Gao, Shaopeng Zhai, Jia Ding, Tianhong Wang, Dayong Liu, Guosheng Ma, Jiang Wu, Dongjun Wan, Junlin Guo, Xinbo Zhang, Jinxia Wu, Yinxu Wang, Ansong Jin, Lei Ma, Huan Yang, Xuexian He, Xiaona Ma, Huijuan Liu, Boya Ma, Ningai Yang, Xiaolin Hou, Ting Xu, Cheng-feng Qin, Huanyu Wang, Peng Xie, Zhenhai Wang

**Affiliations:** a The First Clinical Medical School, Ningxia Medical University, Yinchuan, People’s Republic of China; b Xuanwu Hospital Capital Medical University, Beijing, People’s Republic of China; c Department of Neurology, Lanzhou University Second Hospital, Lanzhou, People’s Republic of China; d Guangzhou Women and Children’s Medical Center, Guangzhou, People’s Republic of China; e Ningxia Medical University, Yinchuan, People’s Republic of China; f Neuro-Intensive Care Unit, The First Affiliated Hospital of Zhengzhou University, Zhengzhou, People’s Republic of China; g West China Hospital of Sichuan University, Chengdu, People’s Republic of China; h Department of Neurology, Xijing Hospital, The Air Force Medical University, Xi’an, People’s Republic of China; i Shandong Provincial Hospital Affiliated to Shandong First Medical University, Jinan, People’s Republic of China; j Neurology Center, General Hospital of Ningxia Medical University, Yinchuan, People’s Republic of China; k The Affiliated Hospital of North Sichuan Medical College, Nanchong, People’s Republic of China; l Meishan People’s Hospital, Meishan, People’s Republic of China; m Chengdu Seventh People’s Hospital, Chengdu, People’s Republic of China; n National Key Laboratory of Intelligent Tracking and Forecasting for Infectious Diseases, National Institute for Viral Disease Control and Prevention, Chinese Center for Disease Control and Prevention, Beijing, People’s Republic of China; o Institute of Medical Sciences, General Hospital of Ningxia Medical University, Yinchuan, People’s Republic of China; p Diagnosis and Treatment Engineering Technology Research Center of Nervous System Diseases of Ningxia, Yinchuan, People’s Republic of China; q Department of Pediatrics, Yibin Hospital, Children's Hospital of Chongqing Medical University, Yibin, People’s Republic of China; r Baoji Central Hospital, Baoji, People’s Republic of China; s The First People’s Hospital of Tianshui, Tianshui, People’s Republic of China; t The First Hospital of Lanzhou University, Lanzhou, People’s Republic of China; u The Affiliated Hospital of Gansu Medical College, Pingliang, People’s Republic of China; v Gansu Provincial People’s Hospital, Lanzhou, People’s Republic of China; w The First People’s Hospital of Longnan, Longnan, People’s Republic of China; x The 940th Hospital of Joint Logistic Support Force of Chinese People’s Liberation Army, Lanzhou, People’s Republic of China; y Qingyang People's Hospital, Qingyang, People’s Republic of China; z The First Affiliated Hospital of Kunming Medical University, Kunming, People’s Republic of China; aa Emergency Center, General Hospital of Ningxia Medical University, Yinchuan, People’s Republic of China; ab Cerebrospinal Fluid Laboratory, General Hospital of Ningxia Medical University, Yinchuan, People’s Republic of China; ac Department of Infectious Diseases, General Hospital of Ningxia Medical University, Yinchuan, People’s Republic of China; ad General Hospital of Ningxia Medical University, Yinchuan, People’s Republic of China; ae State Key Laboratory of Pathogen and Biosecurity, Beijing Institute of Microbiology and Epidemiology, Beijing, People’s Republic of China; af NHC Key Laboratory of Diagnosis and Treatment on Brain Functional Diseases, Chongqing, People’s Republic of China; ag Department of Neurology, the First Affiliated Hospital of Chongqing Medical University, Chongqing, People’s Republic of China

**Keywords:** Japanese encephalitis virus, peripheral nerve injury, clinical classification, endemic, electromyography

## Abstract

Previously, we reported a cohort of Japanese encephalitis (JE) patients with Guillain–Barré syndrome. However, the evidence linking Japanese encephalitis virus (JEV) infection and peripheral nerve injury (PNI) remains limited, especially the epidemiology, clinical presentation, diagnosis, treatment, and outcome significantly differ from traditional JE. We performed a retrospective and multicenter study of 1626 patients with JE recorded in the surveillance system of the Chinese Center for Disease Control and Prevention, spanning the years 2016–2020. Cases were classified into type 1 and type 2 JE based on whether the JE was combined with PNI or not. A comparative analysis was conducted on demographic characteristics, clinical manifestations, imaging findings, electromyography data, laboratory results, and treatment outcomes. Among 1626 laboratory confirmed JE patients, 230 (14%) were type 2 mainly located along the Yellow River in northwest China. In addition to fever, headache, and disturbance of consciousness, type 2 patients experienced acute flaccid paralysis of the limbs, as well as severe respiratory muscle paralysis. These patients presented a greater mean length of stay in hospital (children, 22 years [range, 1–34]; adults, 25 years [range, 0–183]) and intensive care unit (children, 16 years [range, 1–30]; adults, 17 years [range, 0–102]). The mortality rate was higher in type 2 patients (36/230 [16%]) compared to type 1 (67/1396 [5%]). The clinical classification of the diagnosis of JE may play a crucial role in developing a rational treatment strategy, thereby mitigating the severity of the disease and potentially reducing disability and mortality rates among patients.

## Background

Japanese encephalitis virus (JEV) is a neurotropic flavivirus primarily transmitted through the bite of infected Culex tritaeniorhynchus mosquitoes. The first case of Japanese encephalitis (JE) was documented in Japan in 1871, while the first prototype strain was isolated from the cerebral tissue of a deceased patient in 1935 [1]. Worryingly, a study estimated that nearly 100,308 clinical cases of JE and 20,000–30,000 deaths occurred worldwide in 2015 [2]. Over the years, JEV has spread from Asia to Europe, Africa and Australia [3–5]. The initial occurrence of JE in China took place in 1940 [6], prompting the implementation of official surveillance in 1951 [7], wherein all cases were confirmed through laboratory testing. As the seasons progress, JE epidemics manifest in neighbouring cities along the Yangtze and Yellow Rivers in China [8,9]. Interestingly, the transition of JEV genotype from GIII to GIb has resulted in significant alterations in clinical symptoms, treatment approaches, and patient outcomes when compared to previous occurrences [10–12].

Notably, there have been successive reports of outbreaks of Guillain–Barré syndrome (GBS) associated with JEV infection since 1994 in various regions such as southern India and Ningxia in China [13–17]. The outbreaks mentioned above lacked a clearly defined clinical management strategy. Interestingly, previous animal models have provided evidence of a direct causal relationship between the GIb strain of JEV and peripheral nerve injury (PNI) [18,19]. It is indisputable that JEV possesses the capacity to evolve into a global pathogen of significant concern, thereby presenting formidable challenges to both human health and socio-economic stability.

To date, the clinical features, diagnostic and therapeutic measures, and outcomes of PNI associated with JEV infection have not been well described. We reviewed all laboratory-confirmed cases in China from January 2016 to December 2020. In this report, we systematically classify the collected data of JE according to whether PNI cases were incorporated or not.

## Methods

### Inclusion criteria and definitions

The case definition of JE includes three criteria: patients who presented clinical symptoms such as fever, headache, or those who were hospitalized due to manifestations of disturbance of consciousness, respiratory failure, and limb paralysis. Laboratory findings indicated that positive serological or etiological test results were indicative of JEV infection. Epidemiology was defined as living in a region where JE was traditionally endemic and the onset of the disease occurs during the mosquito season; or have travelled to an area where JEV infection was prevalent in the 25 days prior to the onset of illness [20].

Two types of cases lacking laboratory confirmation: clinically diagnosed cases and suspected cases were not included in the study. Cases confirmed before the first week of epidemiology (4 January 2016) were excluded. Instances lacking comprehensive hospitalization data were excluded from this case series. Moreover, these cases were studied according to the World Health Organization JE and Chinese JE diagnostic criteria (WS214–2008) implemented by the International Classification for Standards (ICS 11.020).

### JE surveillance system

The JE surveillance system, maintained by the Chinese Center for Disease Control and Prevention (CDC), necessitates that county-level CDCs promptly conduct individualized investigations of confirmed or suspected cases within 48 h of receiving the report (or within 12 h of an outbreak). Subsequently, the findings are duly recorded in the database and transmitted to the Chinese CDC through the network system [7,20]. JE cases were reported to the Chinese CDC in patients presenting characteristic symptoms, regardless of whether laboratory confirmation was obtained (Table S2).

### Geographic distribution of JE cases

To assess the geographic distribution of the outbreak, we plot the number of JE cases through the nine traditionally endemic regions reported. We calculated the number of PNI cases associated with JE based on the year of onset and clinical characteristics. Figure 2B illustrates the cumulative of reported cases as well as the count of cases confirmed through laboratory testing for each epidemiological week.

### Patients

All patients diagnosed with JE were subsequently categorized into two cohorts: those who exhibited only central nerve injury (CNI) associated with JEV infection, referred to as patients with type 1 JE. And patients with type 2 JE presented CNI and PNI associated with JEV infection (Table 2). Children were defined as individuals under the age of 18, inclusive, while adults were classified as individuals belonging to age groups beyond 18 years.

### Statistical analysis

The study reported the median of the interquartile ranges for the time from JEV infection to hospitalization, the time from onset to disturbance of consciousness, the time from JEV infection to PNI onset, CSF protein levels, and CSF white-cell count. The mean with ranges was used to report the number of days spent in the intensive care unit (ICU) and the total number of days spent in the hospital. The age of the patient was presented as a single mean or means and standard deviations (SDs). Categorical variables were presented as numbers and percentages (N, %). Pairwise deletion was used to handle missing data for all comparisons, and missing data were not included in the denominator for any given descriptive statistic. Differences in characteristics between injury subgroups were assessed using independent sample t tests or Mann–Whitney tests for continuous variables and χ² or Fisher’s exact tests for categorical covariates. Statistical analyzes were performed by SPSS (version 22·0)

## Results

### Cases distribution and demographic characteristics

**Figure 1.**
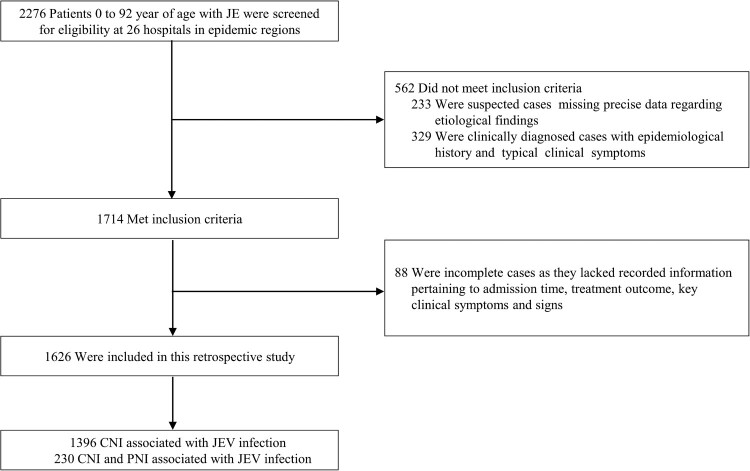
Presentation of surveillance data and infectious outcomes. The confirmation of JE was achieved through the detection of IgM antibody and RT-PCR assay in blood or cerebrospinal fluid samples. Abbreviations: JE, Japanese encephalitis; CNI, central nerve injury; JEV, Japanese encephalitis virus; PNI, peripheral nerve injury.

**Figure 2.**
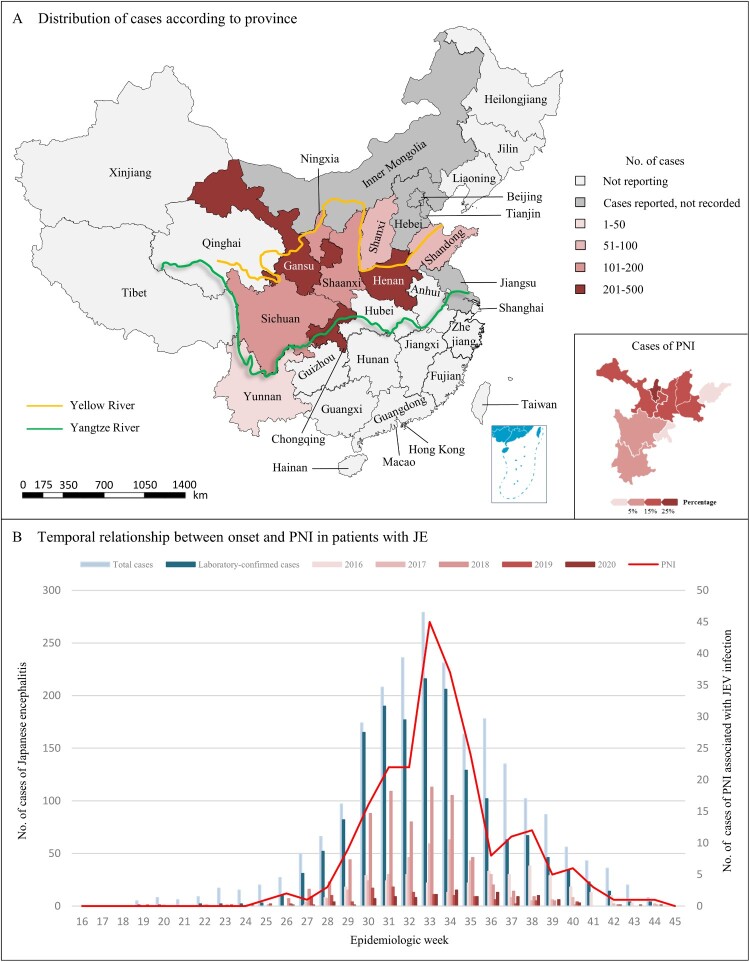
Geographic and temporal representation of cases of JE. Panel A shows the cases of JE and PNI included in this report according to province; cases are only from the reporting hospitals and do not reflect all cases in each province. Panel B shows the time distribution of symptom onset among a subset of patients who were reported to the Chinese CDC surveillance system as having JE from 2016 to 2020. The numbers of total cases and laboratory-confirmed cases are provided. Cases of PNI are confirmed through signs and symptoms as well as clinical and laboratory tests. Abbreviations: PNI, peripheral nerve injury; JE, Japanese encephalitis; JEV, Japanese encephalitis virus.

A comprehensive collection of 1626 cases were obtained from 26 hospitals located in nine reporting regions (Figure 1; Table S2 in the Supplement). The distribution of JE cases is widespread across China, with a predominant concentration in the surrounding regions along the routes of the Yangtze and Yellow Rivers (Figure 2A). The annual distribution of JE cases showed a predominant concentration within the 26th to 42nd epidemiological weeks, and the number of laboratory-confirmed cases as well as PNI cases reached their peak in the 33rd week, indicating a significant surge in disease incidence (Figure 2B). Furthermore, the occurrence of JE cases in 2018 was markedly higher compared to other years from epidemiological week 26th to 35th (Figure 2B). The number of laboratory-confirmed cases in 2018 reached 691 (43%), representing a twofold increase compared to 2017 (343, [21%]) (Figure S1 in the Supplement).

### Clinical features of all JE patients

The incidence of JE was found to be higher in the age groups of 0–15, 46–60, and 61–75 years (Table S1 in the Supplement). The median time from JEV infection to hospitalization was 4 days (IQR 3–6). The most common clinical characteristics included fever (1603, [99%]), headache (1075, [66%]), disturbance of consciousness (1098, [68%]), trouble breathing (309, [19%]), paresthesia (59, [4%]) and convulsion (496, [31%]). Hypermyotonia was observed in 33% (523/1589) of patients, while hyperreflexia was present in 15% (402/1589). Unexpectedly, some patients also developed features of hypomyotonia (245, [15%]) and areflexia or decreased reflexes (281, [18%]). Brain MRI was common in the thalamus (875, [59%]), basal ganglia (337, [23%]), and brainstem (258, [18%]). The lobes, hippocampus, and splenium of the corpus callosum exhibited varying numbers of lesions. Meanwhile, 109 (7%) patients had abnormal EMG results. The median CSF protein level in all patients with JE was 0.47 g/L (IQR 0.1–6.83). JEV-IgM testing was conducted on serum or CSF samples from more than 89% of the patients, and JEV strains were isolated from the CSF of two patients. A total of 566 patients were admitted to the ICU, with 61% requiring mechanical ventilation. The mean length of stay in the ICU was 15 days (range, 0–114). Furthermore, 854 patients (53%) and 493 patients (30%) were administered glucocorticoids and intravenous gamma globulin, respectively (Table 1).

**Table 1.** Clinical findings and laboratory results of 1626 JE patients hospitalized from 2016 to 2020.

Characteristic	No. (%) Patients (*n* = 1626)
Median time from JEV infection to hospitalization, (IQR), d^a^	4 (3–6)
Clinical signs and symptoms	
Fever	1603 (99)
Headache	1075 (66)
Disturbance of consciousness	1098 (68)
Trouble breathing	309 (19)
Paresthesia	59 (4)
Convulsion	496 (31)
Hypermyotonia	523/1589 (33)
Hypomyotonia	245/1589 (15)
Hyperreflexia	402/1589 (25)
Areflexia or decreased reflexes	281/1589 (18)
Brain MRI	
Lobes	232/1477 (16)
Brainstem	258/1477 (18)
Thalamus	875/1477 (59)
Hippocampus	127/1477 (9)
Basal ganglia	337/1477 (23)
Other^b^	74/1477 (5)
EMG	
Abnormal	109 (7)
Incomplete records	29/109 (27)
Results of CSF analysis	
Median protein level, (IQR), g/litre	0.47 (0.1–6.83)
Increased protein level^c^	906/1369 (66)
Median white-cell count per mm^3^ (IQR)	90 (0–1280)
Pathogen detection	
JEV IgM detected in serum	1355/1528 (89)
JEV IgM detected in CSF	1361/1442 (94)
JEV strain isolated from serum	0
JEV strain isolated from CSF^d^	2 (0·1)
Highest level of care	
Ward	1060 (65)
ICU	566 (35)
Mean length of stay in ICU, (range), d	15 (0–114)
Treatment	
Glucocorticoid	854 (53)
Intravenous immune globulin	493 (30)
Mechanical ventilation	343/566 (61)

Abbreviations: JEV, Japanese encephalitis virus; MRI, magnetic resonance imaging; EMG, electromyography; CSF, cerebrospinal fluid; ICU, intensive care unit; IQR, interquartile range; JE, Japanese encephalitis. Percentages may not total 100 because of rounding.

^a^ The onset of symptoms of JE was defined as the first day of onset of fever, headache, dyspnoea, or other neurologic symptoms.

^b^ Excluding other diseases related to the nervous system, MRI lesions in patients with JEV infection also appeared in the splenium of the corpus callosum, unilateral or bilateral insula, cerebellum, and spinal cord.

^c^ The cutoff for defining an increased CSF protein level was 0.45 g per litre.

^d^ From 2016 to 2020, 14 strains of JEV gene I and 1 strain of JEV gene Ib were isolated from mosquitoes (Culex tritaeniorhynchus), aborted piglets, seals and CSF of patients in the above-mentioned epidemic regions. (This data was provided by the arbovirus laboratory of the Chinese Center for Disease Control and Prevention.)

### Characteristics of type 1 and type 2 JE patients

Type 1 and type 2 patients were mostly caused by JEV GIb infection, but there were regional differences in gene clade between the two. The GIb-clade 1 originated in 1992 and mainly concentrated in the Yangtze River basin in southern China (such as Yunnan, Chongqing, Sichuan), while the GIb-clade 2 belonged to the emerging clade, originated in 2016. It is mainly concentrated in the basins along the Yellow River in northwest China (such as Ningxia, Gansu and Shaanxi) [11,21]. The prevalence of adults with both types of JE was significantly higher compared to the traditional JE population, while patients with type 2 JE tended to be relatively older than those with type 1 JE (Table 2; Table S3 in the Supplement).

**Table 2.** Neurological features in children and adults associated with JE in 1626 patients.

Characteristic	No. (%)				*p* Value^a^
	Type 1 JE		Type 2 JE		
	Children	Adults	Children	Adults	
	(*n* = 514)	(*n* = 882)	(*n* = 12)	(*n* = 218)	
Demographics					
Age, mean (SD; range), y	8 (5; 0.1–18)	53 (16; 19–92)	13 (5; 2–18)	55 (16; 19–84)	<.0001
Male sex	318 (62)	427 (48)	5 (42)	119 (55)	< .0001
Clinical signs and symptoms					
Fever	513 (99)	864 (98)	12 (100)	214 (98)	.0270
Limb weakness followed by fever	0	0	1 (8)	74 (34)	< .0001
Disturbance of consciousness	439 (85)	462 (52)	9 (75)	188 (86)	.0260
Median time from onset to disturbance of consciousness, (IQR), d	4 (2–7)	3 (1–5)	3 (1–6)	5 (2–8)	< .0001
Trouble breathing	56 (11)	91 (10)	9 (75)	153 (70)	< .0001
Median time from onset to trouble breathing, (IQR), d	5 (3–9)	4 (0–6)	2 (1–4)	3 (1–5)	.0020
Facial palsy					
Unilateral facial palsy	0	0	0	26/34 (77%)	NA
Bilateral facial palsy	0	0	0	8/34 (24%)	NA
Blood pressure decreased	0	0	0	6 (3%)	NA
Acute flaccid paralysis^b^	NA	NA	12 (100)	218 (100)	< .0001
Symmetric upper and lower extremity muscle weakness	NA	NA	12 (100)	218 (100)	< .0001
Hypomyotonia	NA	NA	12 (100)	218 (100)	< .0001
Areflexia or decreased reflexes	NA	NA	12 (100)	218 (100)	< .0001
Median time from JEV infection to onset of PNI, (IQR), d^c^	NA	NA	2 (1–4)	3 (0–6)	.0030
Brain MRI					
Corpus callosum (splenium)	3/465 (1)	8/795 (1)	0	19/205 (9)	< .0001
Unilateral or bilateral insula	5/465 (1)	14/795 (2)	0	0	< .0001
Unilateral or bilateral cerebellum	5/465 (1)	13/795 (2)	0	0	< .0001
Spinal cord	0	7/795 (1)	0	0	NA
CSF albuminocytologic dissociation	NA	NA	4/9 (44)	129/205 (63)	< .0001
Abnormal EMG	NA	NA	2/2 (100)	78/107 (73)	< .0001
Simple MCV decreased	NA	NA	2 (100)	51 (65)	< .0001
Simple SCV decreased	NA	NA	0	3 (4)	NA
Both MCV and SCV decreased	NA	NA	0	24 (31)	NA
Decreased CMAP amplitude	NA	NA	0	50 (64)	NA
Disappeared F-waves or H-reflections	NA	NA	0	28 (36)	NA
Treatment					
Intravenous immune globulin	281/345 (82)	125/521 (24)	4/8 (50)	83/175 (47)	< .0001
ICU admission	137 (27)	277 (31)	4 (33)	148 (68)	.0480
Mechanical ventilation	72/137 (53)	172/277 (62)	4/4 (100)	95/148 (64)	.0030
Mean length of stay, (range), d					
In hospital	20 (1–129)	18 (0–146)	22 (1–34)	25 (0-183)	< .0001
In ICU	14 (1–114)	12 (0–68)	16 (1–30)	17 (0–102)	< .0001
Median duration of mechanical ventilation, (IQR), d	6 (3–10)	10 (6–16)	11 (7–17)	16 (11–29)	.0090
Percentage of hospitalization time spent in ICU	60.2	64.5	61.1	69.9	< .0001
Outcome					
Discharged alive	499 (97)	830 (94)	9 (75)	185 (85)	.3280
Died	15 (3)	52 (6)	3 (25)	33 (15)	< .0001
No administered immune globulin	11/15 (73)	40/52 (77)	3/3 (100)	29/31 (94)	< .0001
Follow-up					
Limb muscle weakness	2/62 (3)	34/111 (31)	3/3 (100)	79/141 (56)	< .0001
Limb muscle atrophy	0	0	1/3 (33)	26/141 (18)	.0060

Missing data were excluded from each analysis, so the denominator is different between variables.

Abbreviations: JE, Japanese encephalitis; SD, standard deviation, JEV, Japanese encephalitis virus; PNI, peripheral nerve injury; MRI, magnetic resonance imaging; CSF, cerebrospinal fluid; EMG, electromyography; MCV, motor conduction velocities; SCV, sensory conduction velocities; CMAP, compound muscle action potential; ICU, intensive care unit. Percentages may not total 100 because of rounding.

^a^ *p* values reflect comparisons between all four groups. Statistical testing was done using either Mann-Whitney for continuous variables or χ² for categorical variables.

^b^ Acute flaccid paralysis is the main clinical feature of peripheral nerve injury. It is characterized by decreased muscle strength, hypomyotonia, areflexia or decreased reflexes, and negative pyramidal signs.

^c^ The onset of symptoms of the PNI was defined as the first day of onset of limb weakness, sensory dysfunction, facial paralysis, hypomyotonia, areflexia or decreased reflexes, etc.

The presence of varying degrees of fever has been observed in over 98% of patients diagnosed with both type 1 and type 2 JE, but fever was not the first symptom in all patients with JE. Specifically, in 33% of type 2 patients, limb weakness precedes the onset of fever. The absence of comparable symptoms was observed in type 1 JE. Some patients had disturbance of consciousness, however, the median duration of disturbance of consciousness in type 2 adults were 5 days (IQR 2–8), which was longer than type 1 patients (3 days, IQR [1–5]) (Table 2). Notably, trouble breathing was observed in a subset of patients diagnosed with type 1, with 56 (11%) children and 91 (10%) adults experiencing this symptom. However, the duration of the trouble breathing was brief and most patients recovered within 1–2 days. Type 2 patients appeared trouble breathing in 9 (75%) children and 153 (70%) adults, the duration of this symptom was prolonged and usually ranging from 7 to 10 days. Furthermore, 34 (16%) of the type 2 patients presented unilateral or bilateral facial palsy, while autonomic nerve damage was observed in 6 adult patients (Table 2).

The presentation of unilateral limb paralysis for a brief duration, accompanied by hyperreflexia and positive pyramidal signs, was observed in certain patients diagnosed with type 1 JE. Conversely, type 2 JE consistently presented with flaccid paralysis, predominantly characterized by symmetrical muscle weakness of the upper and lower limbs, hypomyotonia, areflexia or decreased reflexes, and evident limb muscle atrophy. The median time from JEV infection to the onset of signs and symptoms of PNI was 2 (IQR 1–4) and 3 (IQR 0–6) days in children and adults, respectively. The brain MRI revealed involvement of the splenium of the corpus callosum in 9% of type 2 patients, and only 1% of type 1 patients, in addition to the commonly affected thalamus, cerebral peduncle, and basal ganglia lesions. Interestingly, type 1 patients exhibited lesions in the unilateral or bilateral hippocampus, cerebellum, and spinal cord, whereas such lesions were not observed in type 2 patients (Table 2).

A total of 133 (62%) patients with type 2 exhibited albuminocytologic dissociation in the CSF, while 82 patients (75%) met Brighton criteria Level 1 [22]. The EMG of 80 patients with type 2 JE revealed abnormalities, characterized by a decrease in motor conduction velocity (MCV), sensory conduction velocity (SCV) and compound muscle action potential amplitude (CMAP). The diagnosis of JEV-associated GBS (JEV-GBS) was confirmed in 109 patients, accounting for 47% of the type 2 JE cases. The EMG diagnosis revealed 12 cases (11%) of acute inflammatory demyelinating neuropathy (AIDP), 70 cases (64%) of acute motor axonal neuropathy (AMAN), 24 cases (22%) of acute motor-sensory axonal neuropathy (AMSAN), and 3 cases (3%) of acute sensory neuropathy (ASN). (Table S4, Table S5 and Table S6 in the Supplement).

Surprisingly, patients with type 1 and type 2 JE had a higher utilization rate of intravenous immune globulin at 47% and 48%, respectively, despite differences in efficacy and outcome. Among type 1 cases, 414 (30%) were admitted to the ICU and 244 (59%) necessitated mechanical ventilation, with a short median duration of mechanical ventilation lasting 6 days (IQR 3–10). However, 152 (66%) type 2 were admitted to the ICU, and 65% received mechanical ventilation. Mean length of stay, length of ICU stay, median length of mechanical ventilation treatment, and percentage of ICU stay in type 2 patients were significantly higher than type 1 (Table 2). Moreover, the mortality for type 2 was more than double that observed in type 1. These findings suggest a higher incidence of critical illness or increased mortality rates among individuals with PNI associated with JEV infection. Sequelae of diverse magnitudes were observed among the patients in the follow-up, including limb muscle weakness (57%) and muscle atrophy (19%). The majority of these cases pertain to type 2 JE, potentially indicating a correlation with PNI and the absence of early administration of immunotherapy (Table 2).

## Discussion

The 1626 patients with JE came from most of the regions along the Yellow and Yangtze Rivers, which span most of the northern and southern parts of China from west to east, and are the regions with high incidence of JE (Figure 2A). The underlying factor for this phenomenon could potentially be attributed to the concentrated presence of Culex tritaeniorhynchus, which accounts for 56% of the overall mosquito species, as well as alterations in the ecological environment within China (Figure S2A in the Supplement) [8,9].

The increased chance of JEV infection due to factors such as temperature, humidity and wind speed appears to be extremely similar to the geographical distribution of dengue cases in China in 2019 [23]. And those who regularly feed their livestock are most at risk [24,25]. This finding suggests a strong correlation between the prevalence of JEV infection and the geographical context, with more cases in adults than in children (Table 2; Figure S2B in the Supplement), and those certain patients potentially associated with unvaccinated and reduced levels of serum neutralizing antibody levels after vaccination, despite a 15% immunization rate among the patients [8,26,27]. From another perspective, the JE vaccination for adults should be of great concern. In particular, those who initially live in a non-epidemic region should receive vaccination before travelling to a region with a high incidence of JE.

In previous study, we confirmed that GBS was associated with JEV GIb [15]. Examination of the temporal and spatial correlation of JEV GI transmission revealed that the isolated viral strain belonged to the GIb clade 2 epidemic cluster, presenting a higher prevalence in northern China compared to southern China [21]. Until now, the majority of research has primarily focused on the attributes and pathogenesis of CNI following JEV infection [28–31]. However, there is no complete epidemiological and clinical data to establish an association between JEV and PNI. The previous viewpoints were that motor dysfunction caused by JE primarily resulted from central nervous system injury. However, our understanding of PNI associated with JEV infection remains vague and overlooked. Given the incomplete comprehension of the pathogenesis underlying PNI associated with JEV infection, reliable animal models are imperative for investigating its molecular mechanisms. Recently, we have effectively developed a mouse model that substantiates the occurrence of myelin sheath and axonal damage at eight and thirteen days, respectively, following JEV GIb infection [19]. Therefore, a direct causal connection between JEV and PNI has been established.

Although a few rare cases of JE with myelitis and flaccid paralysis have been reported [32], we have not found such special cases in our limited records. It is also possible that clinicians incorrectly attributed flaccid paralysis to myelopathy. In the cohort of cases collected, type 2 patients experienced a prolonged duration of consciousness disturbance and trouble breathing compared to type 1. This discrepancy may be attributed to the presence of acute respiratory failure resulting from respiratory muscle paralysis accompanied by central respiratory failure, which was predominantly observed in type 2. Furthermore, it is worth noting that corpus callosum damage may serve as an exacerbating factor in some cases.

The study found that type 2 patients had a significantly longer average hospital stays and higher mortality rate compared to type 1. After discharge, 141 patients experienced persistent limb muscle weakness and muscle atrophy. 53% of patients did not receive early EMG and immunotherapy, which could potentially contribute to their unfavourable prognosis. An escalation in the number of patients presenting with GBS has been observed by numerous neurologists amidst the JE epidemic (Figure S3 in the Supplement). Consequently, there exists a pressing requirement for the establishment of international guidelines pertaining to various aspects including the diagnosis, treatment protocols, and overall management of JEV-GBS. Particularly, these guidelines are crucial in facilitating the determination of diagnostic measures for JEV-GBS, as well as establishing the optimal timing for initiating and repeating treatment interventions for GBS patients.

**Figure 3.**
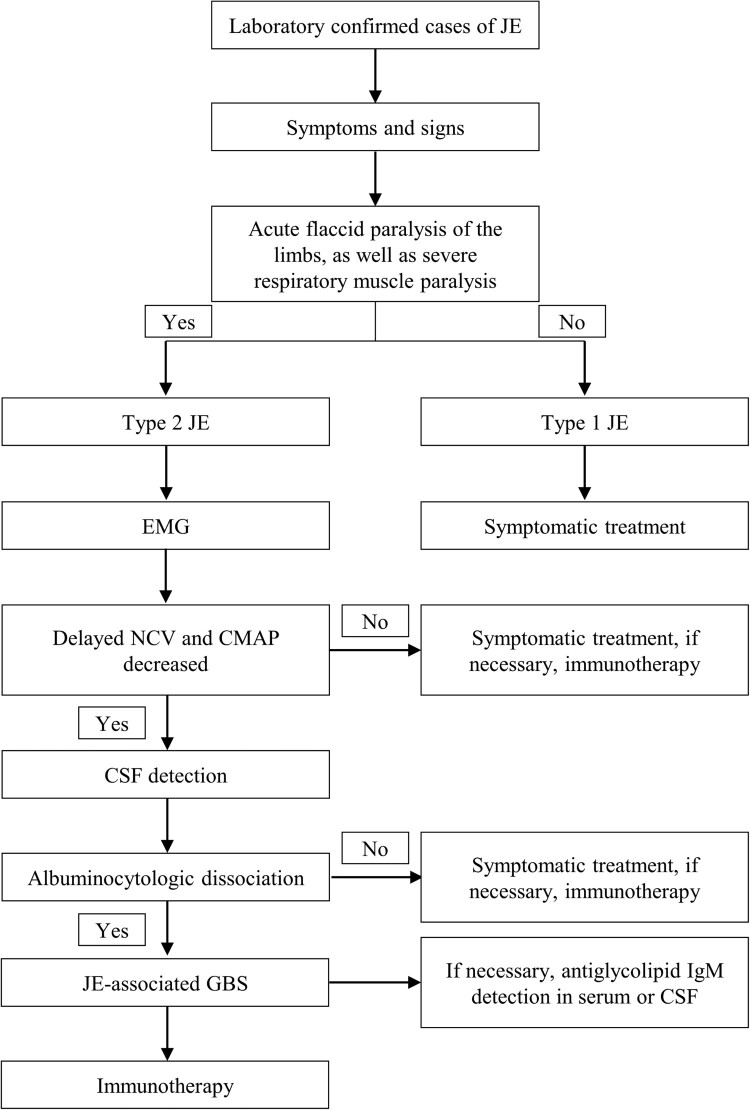
Suggestions for diagnosis and treatment of JEV infection. Abbreviations: JE, Japanese encephalitis; EMG, electromyography; NCV, nerve conduction velocity; CMAP, compound muscle action potential; CSF, cerebrospinal fluid; GBS, Guillain–Barré syndrome.

We suggest a comprehensive assessment of patients with JEV infection, including a meticulous evaluation of clinical symptoms and neurological physical examination, as well as the implementation of brain MRI, electroencephalogram examination, CSF analysis, and other supplementary diagnostic tests during treatment. EMG is recommended for the onset of the disease in patients experiencing severe respiratory muscle paralysis and limb weakness. Additionally, blood or CSF anti-ganglioside antibody testing should be considered if deemed necessary. These results are beneficial to the clinical classification of JE and the development of appropriate treatment strategy, which may reduce the duration of hospitalization, and enhance the prognosis (Figure 3).

The migration of the JEV and the subsequent mutation of its genotype have raised significant concerns because of the possibility that its conventional pathogenic properties could lead to novel organ damage. Consequently, the imperative of disease surveillance becomes paramount, as the emergence of new disease patterns holds the potential to instigate a worldwide public health crisis.

## Supplementary Material

Supplementary_Appendices

## Data Availability

The data that support the findings of this study are available from the corresponding author, [Zhenhai Wang: wangzhenhai1968@163.com], upon reasonable request.

## References

[1] Solomon T, Ni H, Beasley DWC, et al. Origin and evolution of Japanese encephalitis virus in Southeast Asia. J Virol. 2003;77:3091–3098. doi:10.1128/JVI.77.5.3091-3098.2003 12584335 PMC149749

[2] Quan TM, Thao TTN, Duy NM, et al. Estimates of the global burden of Japanese encephalitis and the impact of vaccination from 2000-2015. Elife. 2020;9:e51027. doi:10.7554/eLife.51027 32450946 PMC7282807

[3] van den Hurk AF, Ritchie SA, Mackenzie JS. Ecology and geographical expansion of Japanese encephalitis virus. Annu Rev Entomol. 2009;54:17–35. doi:10.1146/annurev.ento.54.110807.090510 19067628

[4] Gao X, Liu H, Li X, et al. Changing geographic distribution of Japanese encephalitis virus genotypes, 1935-2017. Vector Borne Zoonotic Dis. 2019;19:35–44. doi:10.1089/vbz.2018.2291 30207876

[5] Simon-Loriere E, Faye O, Prot M, et al. Autochthonous Japanese encephalitis with yellow fever coinfection in Africa. N Engl J Med. 2017;376:1483–1485. doi:10.1056/NEJMc1701600 28402771

[6] Zheng Y, Li M, Wang H, et al. Japanese encephalitis and Japanese encephalitis virus in mainland China. Rev Med Virol. 2012;22:301–322. doi:10.1002/rmv.1710 22407526

[7] Chen XJ, Wang HY, Li XL, et al. Japanese encephalitis in China in the period of 1950-2018: from discovery to control. Biomed Environ Sci. 2021;34:175–183. 33766213 10.3967/bes2021.024

[8] Liu B, Gao X, Ma J, et al. Influence of host and environmental factors on the distribution of the Japanese encephalitis vector culex tritaeniorhynchus in China. Int J Environ Res Public Health. 2018;15:1848. doi:10.3390/ijerph15091848 30150565 PMC6165309

[9] Fang Y, Li X-S, Zhang W, et al. Molecular epidemiology of mosquito-borne viruses at the China-Myanmar border: discovery of a potential epidemic focus of Japanese encephalitis. Infect Dis Poverty. 2021;10:57. doi:10.1186/s40249-021-00838-z 33902684 PMC8073957

[10] Wang L, Fu S, Zhang H, et al. Identification and isolation of genotype-I Japanese encephalitis virus from encephalitis patients. Virol J. 2010;7:345. doi:10.1186/1743-422X-7-345 21108846 PMC3002311

[11] Liu W, Fu S, Ma X, et al. An outbreak of Japanese encephalitis caused by genotype Ib Japanese encephalitis virus in China, 2018: A laboratory and field investigation. PLoS Negl Trop Dis. 2020;14:e0008312. 32453787 10.1371/journal.pntd.0008312 PMC7274457

[12] Gao X, Liu H, Li X, et al. Changing geographic distribution of Japanese encephalitis virus genotypes, 1935-2017. Vector Borne Zoonotic Dis. 2019;19:35–44. doi:10.1089/vbz.2018.2291 30207876

[13] Ravi V, Taly AB, Shankar SK, et al. Association of Japanese encephalitis virus infection with Guillain-Barré syndrome in endemic areas of south India. Acta Neurol Scand. 1994;90:67–72. doi:10.1111/j.1600-0404.1994.tb02681.x 7941960

[14] Bandyopadhyay D, Ganesan V, Choudhury C, et al. Two uncommon causes of Guillain-Barré syndrome: hepatitis E and Japanese encephalitis. Case Rep Neurol Med. 2015;2015:759495. 26798531 10.1155/2015/759495 PMC4700154

[15] Wang G, Li H, Yang X, et al. Guillain-Barré syndrome associated with JEV infection. N Engl J Med. 2020;383:1188–1190. doi:10.1056/NEJMc1916977 32937054

[16] Liu S, Wang J, Yang J, et al. The underlying mechanism of Guillain-Barré syndrome in a young patient suffered from Japanese encephalitis virus infection: a case report. Virol J. 2022;19:139. doi:10.1186/s12985-022-01870-7 36050705 PMC9434870

[17] Zang Q, Wang Y, Guo J, et al. Treatment of severe Japanese encephalitis complicated with hashimoto's thyroiditis and Guillain-Barré syndrome with protein A immunoadsorption: a case report. Front Immunol. 2022;12:807937. doi:10.3389/fimmu.2021.807937 35069593 PMC8777188

[18] Wang X, Wang G, Yang H, et al. A mouse model of peripheral nerve injury induced by Japanese encephalitis virus. PLoS Negl Trop Dis. 2022;16:e0010961. 36441775 10.1371/journal.pntd.0010961 PMC9731479

[19] Yang H, Wang X, Wang Z, et al. Peripheral nerve injury induced by Japanese encephalitis virus in C57BL/6 mouse. J Virol. 2023;97:e0165822. 37071015 10.1128/jvi.01658-22 PMC10231255

[20] The interim measures for the prevention and treatment of infectious diseases. Beijing Municipal Daily. 1951; 2.

[21] Li F, Feng Y, Wang G, et al. Tracing the spatiotemporal phylodynamics of Japanese encephalitis virus genotype I throughout Asia and the western pacific. PLoS Negl Trop Dis. 2023;17:e0011192. 37053286 10.1371/journal.pntd.0011192 PMC10128984

[22] Fokke C, van den Berg B, Drenthen J, et al. Diagnosis of Guillain-Barré syndrome and validation of Brighton criteria. Brain. 2014;137:33–43. doi:10.1093/brain/awt285 24163275

[23] Yue Y, Liu X, Ren D, et al. Spatial dynamics of dengue fever in mainland China, 2019. Int J Environ Res Public Health. 2021;18:2855. doi:10.3390/ijerph18062855 33799640 PMC7999437

[24] Mansfield KL, Hernández-Triana LM, Banyard AC, et al. Japanese encephalitis virus infection, diagnosis and control in domestic animals. Vet Microbiol. 2017;201:85–92. doi:10.1016/j.vetmic.2017.01.014 28284628

[25] Xiao C, Wang X, Cui G, et al. Possible pathogenicity of Japanese encephalitis virus in newly hatched domestic ducklings. Vet Microbiol. 2018;227:8–11. doi:10.1016/j.vetmic.2018.10.016 30473356

[26] Pearce JC, Learoyd TP, Langendor BJ, et al. Japanese encephalitis: the vectors, ecology and potential for expansion. J Travel Med. 2018;25:S16–S26. doi:10.1093/jtm/tay009 29718435

[27] Misra UK, Kalita J. Overview: Japanese encephalitis. Prog Neurobiol. 2010;91:108–120. doi:10.1016/j.pneurobio.2010.01.008 20132860

[28] Waller C, Tiemensma M, Currie BJ, et al. Japanese encephalitis in Australia - a sentinel case. N Engl J Med. 2022; 387:661–662. doi:10.1056/NEJMc2207004 36070717

[29] Ashraf U, Ding Z, Deng S, et al. Pathogenicity and virulence of Japanese encephalitis virus: neuroinflammation and neuronal cell damage. Virulence. 2021;12:968–980. doi:10.1080/21505594.2021.1899674 33724154 PMC7971234

[30] Yadav P, Chakraborty P, Jha NK, et al. Molecular mechanism and role of Japanese encephalitis virus infection in central nervous system-mediated diseases. Viruses. 2022;14:2686. doi:10.3390/v14122686 36560690 PMC9781168

[31] Hsieh JT, Rathore APS, Soundarajan G, et al. Japanese encephalitis virus neuropenetrance is driven by mast cell chymase. Nat Commun. 2019;10:706. doi:10.1038/s41467-019-08641-z 30742008 PMC6370868

[32] Grewe S, Gliem M, Abrar DB, et al. Myelitis with flaccid paralysis due to Japanese encephalitis: case report and review of the literature. Infection. 2022;50:1597–1603. doi:10.1007/s15010-022-01815-w 35396695 PMC8993587

