# Supplementary material for: Peripheral nerve injury associated with JEV infection in high endemic regions, 2016–2020: a multicenter retrospective study in China
Source: Emerg Microbes Infect. 2024 Apr 5;13(1):2337677. doi: 10.1080/22221751.2024.2337677 (PMC11036900; doi:10.1080/22221751.2024.2337677)
Supplement: Supplementary_Appendices [file TEMI_A_2337677_SM0056.docx]

**Supplementary Appendices**

**Table of contents**

| Page | Content |
| --- | --- |
| 2 | Table of contents |
| 3-5 | Participating sites and personnel |
| 6 | Supplementary methods |
| 7 | Supplementary tables  Table S1. Demographic characteristic of the patients according to JEV infection status |
| 8 | Table S2. Number of laboratory-confirmed cases of JE recorded by the Chinese CDC |
| 8 | Table S3. Demographic data of 1626 patients with JE |
| 9-10 | Table S4. Electrophysiological results of 80 patients with PNI associated with JEV infection |
| 11 | Table S5. Motor and sensory nerve conduction outcomes in 80 patients with PNI associated with JEV infection |
| 12-13 | Table S6. Neurological features of GBS patients associated with JEV infection |
| 14 | Supplementary figures  Figure S1. Patients with symptoms of JE, including laboratory-confirmed cases and PNI cases in each epidemiologic year (January 4, 2016–December 27, 2020) |
| 14 | Figure S2. The distribution of cases of PNI associated with JEV infection and investigation of mosquitoes in epidemic regions |
| 15 | Figure S3. Temporal relationship between onset and GBS in patients with Japanese encephalitis |
| 16-17 | Figure S4. The research project agreement of this study |
| 18-19 | Form - Demographic Information, Clinical Features and Laboratory Text Results of JE Patients |
| 20 | References |

**Participating sites and personnel**

School of Clinical Medicine, Ningxia Medical University, Yinchuan, China.

Investigators: Guowei Wang, M.M, Tingting Xuan, M.M, Shuting Liu, M.S, Xiaocong Li, M.M, Huan Yang, M.M, Liping Yang, M.M, Kaichun Shen, M.M, Yanping Yuan, M.M, Na Zhang, M.M.

Institute of Medical Sciences, General Hospital of Ningxia Medical University;

Diagnosis and Treatment Engineering Technology Research Center of Nervous System Diseases of Ningxia, Yinchuan, China.

Investigators: Zhenhai Wang, M.D, Tingting Yang, M.Sc, Ningai Yang, M.M, Xiaona Ma, M.B.

Neurology Center, General Hospital of Ningxia Medical University, Yinchuan, China.

Investigators: Zhenhai Wang, M.D, Haining Li, M.D, Lufei Shao, M.M, Boya Ma, M.M, Xiaolin Hou, M.M, Ting Xu, M.M.

NHC Key Laboratory of Diagnosis and Treatment on Brain Functional Diseases; Department of Neurology, the First Affiliated Hospital of Chongqing Medical University, Chongqing, China.

Investigator: Peng Xie, M.D.

State Key Laboratory of Pathogen and Biosecurity, Beijing Institute of Microbiology and Epidemiology, Beijing, China

Investigator: Chengfeng Qin, Ph.D.

Department of Arbovirus, National Institute for Viral Disease Control and Prevention, Chinese Center for Disease Control and Prevention; State Key Laboratory for Infectious Disease Prevention and Control, Chinese Center for Disease Control and Prevention, Beijing, China.

Investigators: Huanyu Wang, Ph.D, Shihong Fu, B.S, Fan Li, Ph.D.

Xuanwu Hospital Capital Medical University, Beijing, China.

Investigator: Lianmei Zhong, M.D.

Guangzhou Women and Children’s Medical Center, Guangzhou, China.

Investigator: Juan Zhou, M.D.

The Second Hospital of Lanzhou University, Lanzhou, China.

Investigators: Manxia Wang, M.D, Xiaoling Li, M.D, Shaopeng Zhai, M.M.

Neuro-Intensive Care Unit of the First Affiliated Hospital of Zhengzhou University, Zhengzhou, China. Investigator: Wang Miao, M.D.

West China Hospital, Sichuan University, Chengdu, China.

Investigator: Leilei Li, M.D.

Meishan People’s Hospital, Meishan, China.

Investigator: Liuqing Xie, M.M.

Chengdu Seventh People’s Hospital, Chengdu, China.

Investigator: Min Xie, M.M.

The First People’s Hospital of Yibin, Yibin, China.

Investigators: Mingfang Shi, M.B, Jinxia Wu, M.B, Li Gu, M.D.

Department of Neurology, the First Affiliated Hospital, the Air Force Military Medical University, Xi’an, China.

Investigator: Yonghong Liu, M.D, Xinbo Zhang, M.M.

The Affiliated Hospital of North Sichuan Medical College, Nanchong, China.

Investigators: Xiaoming Wang, M.M, Yinxu Wang, M.M.

The First Affiliated Hospital of Kunming Medical University, Kunming, China.

Investigators: Ansong Jin, M.M.

Shandong Provincial Hospital Affiliated to Shandong First Medical University, Jinan, China.

Investigator: Shougang Guo, M.D.

The 940th Hospital of Joint Logistic Support Force of Chinese People’s Liberation Army, Lanzhou, China.

Investigator: Dongjun Wan, Ph.D.

The First Hospital of Lanzhou University, Lanzhou, China.

Investigator: Tianhong Wang, M.D.

The First People’s Hospital of Tianshui, Tianshui, China.

Investigator: Jia Ding, M.B.

The Affiliated Hospital of Gansu Medical College, Pingliang, China.

Investigator: Dayong Liu, M.B.

Gansu Provincial People’s Hospital, Lanzhou, China.

Investigator: Guosheng Ma, M.D.

The First People’s Hospital of Longnan, Longnan, China.

Investigator: Jiang Wu, M.M.

Qingyang People's Hospital, Qingyang, China.

Investigator: Junlin Guo, M.B.

Baoji Central Hospital, Baoji, China.

Investigator: Li Gao, M.M.

Emergency Center, General Hospital of Ningxia Medical University, Yinchuan, China.

Investigator: Lei Ma, M.M.

Department of Infectious Diseases, General Hospital of Ningxia Medical University, Yinchuan, China.

Investigator: Huijuan Liu, M.M.

Cerebrospinal Fluid Laboratory, General Hospital of Ningxia Medical University, Yinchuan, China. Investigator: Xuexian He, M.M.

**Supplementary methods**

All cases of JE that were confirmed in the laboratory were obtained from the surveillance system of the Chinese CDC (see Table S2). We strategically selected nine provinces with a high incidence of JE in traditional epidemic regions as locations for collecting cases. These provinces are predominantly situated along the banks of the Yangtze River and the Yellow River, which are conducive to mosquito breeding. Moreover, the presence of a substantial livestock population in the local rural areas creates a favorable environment for JEV transmission.

Laboratory-confirmed JE is defined as the manifestation of clinical symptoms along with one of the subsequent test outcomes: (1) individuals who have not been administered the JEV vaccine within a month, and exhibit positive anti-JEV IgM antibodies in their blood or CSF; (2) The convalescent serum exhibits a significantly elevated level of anti-JEV IgG antibody or JEV neutralizing antibody titer, surpassing four times the amount observed during the acute phase. Alternatively, the presence of anti-JEV IgM or IgG antibodies is absent during the acute phase but becomes positive during the recovery phase. (3) The detection of JEV antigen or specific nucleic acid in tissues, blood, or other body fluids is achieved through direct immunofluorescence or polymerase chain reaction. (4) JEV isolation was successfully accomplished from CSF, brain tissue, and serum samples.

This retrospective study established collaborative agreements with hospitals that satisfied the inclusion criteria for cases in nine provinces (Figure S4). Each hospital willingly joined the study as a member of the group, and meticulous medical records were entered into a standardized format by dedicated investigators at the participating locations. Cases identified for submission include the intensive care unit, the neurology center, the department of infectious disease and the pediatrics. The dataset encompassed various parameters such as demographics, underlying health conditions, clinical symptoms and signs, imaging and electrophysiological outcomes, laboratory tests, diagnostic findings, treatment, and post-discharge follow-up. Through careful analysis of clinical features and laboratory results, we ruled out the possibility of other bacterial or viral infections in these patients.

To address duplicate reports, we excluded any overlapping reports pertaining to this particular study. Investigators reported a case of JE with GBS and Hashimoto's thyroiditis.^1^ But this extremely uncommon case is not within the time frame collected in this study. Another possible overlap could not be verified. Investigators assessed the effectiveness of combination ganciclovir, methylprednisolone, and immunoglobulin therapy in reducing cognitive impairment and mortality in 31 patients with JE.^2^ We compared demographic information, clinical characteristics, and treatment, and excluded these cases even though there was no duplication of data.

**Supplementary tables**

| Table S1. Demographic characteristic of the patients according to JEV infection status. |
| --- |
| No. (%) |
| Age |
| Mean, (SD), y 38 |
| Distribution |
| 0-15 y 475 (29) |
| 16-30 y 171 (11) |
| 31-45 y 184 (11) |
| 46-60 y 368 (23) |
| 61-75 y 375 (23) |
| >76 y 53 (3) |
| Sex |
| Male 869 (53) |
| Female 757 (47) |
| Occupation |
| Peasant 670 (41) |
| Student 335 (21) |
| Other^a^ 621 (38) |
| JEV vaccination history 236 (15) |
| Residence or work history in the ER^b^ 1325 (82) |
| Medical history |
| Hypertension 304 (19) |
| Diabetes mellitus 65 (4) |
| Coronary disease 38 (2) |
| Drug allergy 22 (1) |
| Abbreviations: JEV, Japanese encephalitis virus; ER, epidemic regions. Percentages may not total 100 because of rounding.  ^a^ Teachers, industrial workers, businessmen, individual operator and the unemployed were recorded in epidemiological data.  ^b^ People who lived and worked in the ER for a long time or traveled and visited the ER for a short period of time (i.e. people who had been in the ER during JEV infection) were classified as having an epidemiological link. |

| Table S2. Number of laboratory-confirmed cases of JE recorded by the Chinese CDC. |
| --- |
| Provinces Cases Monitored (CM) Cases Collected (CC) CC/CM (%) |
| Beijing 42 —^a^ — |
| Gansu 839 470 56 |
| Hebei 83 — — |
| Henan 425 277 65 |
| Jiangsu 28 — — |
| Ningxia 170 163 96 |
| Chongqing 289 279 97 |
| Inner Mongolia 8 — — |
| Shandong 298 77 26 |
| Shanxi 197 58 29 |
| Tianjin 4 — — |
| Shaanxi 703 101 14 |
| Sichuan 450 170 38 |
| Yunnan 403 31 8 |
| Abbreviations: JE, Japanese encephalitis; CDC, Centers for Disease Control and Prevention.  ^a^ We collected data from provinces with a traditionally high incidence of JE with more than 100 cases between 2016 and 2020. |

| Table S3. Demographic data of 1626 patients with JE. |
| --- |
| Provinces Male Female Adult Children  (n = 869) (n = 757) (n = 1100) (n = 526) |
| Gansu 235/470 (50) 235/470 (50) 437/470 (93) 33/470 (7) |
| Henan 133/277 (48) 144/277 (52) 222/277 (80) 55/277 (20) |
| Ningxia 92/163 (56) 71/163 (44) 157/163 (96) 6/163 (4) |
| Chongqing 174/279 (62) 105/279 (38) 0 279/279 (100) |
| Shandong 40/77 (52) 37/77 (48) 47/77 (61) 30/77 (39) |
| Shanxi 32/58 (55) 26/58 (45) 48/58 (83) 10/58 (17) |
| Shaanxi 5/101 (45) 56/101 (55) 78/101 (77) 23/101 (23) |
| Sichuan 104/170 (61) 66/170 (39) 87/170 (51) 83/170 (49) |
| Yunnan 14/31 (45) 17/31 (55) 20/31 (65) 11/31 (36) |
| Abbreviations: JE, Japanese encephalitis. Percentages may not total 100 because of rounding. |


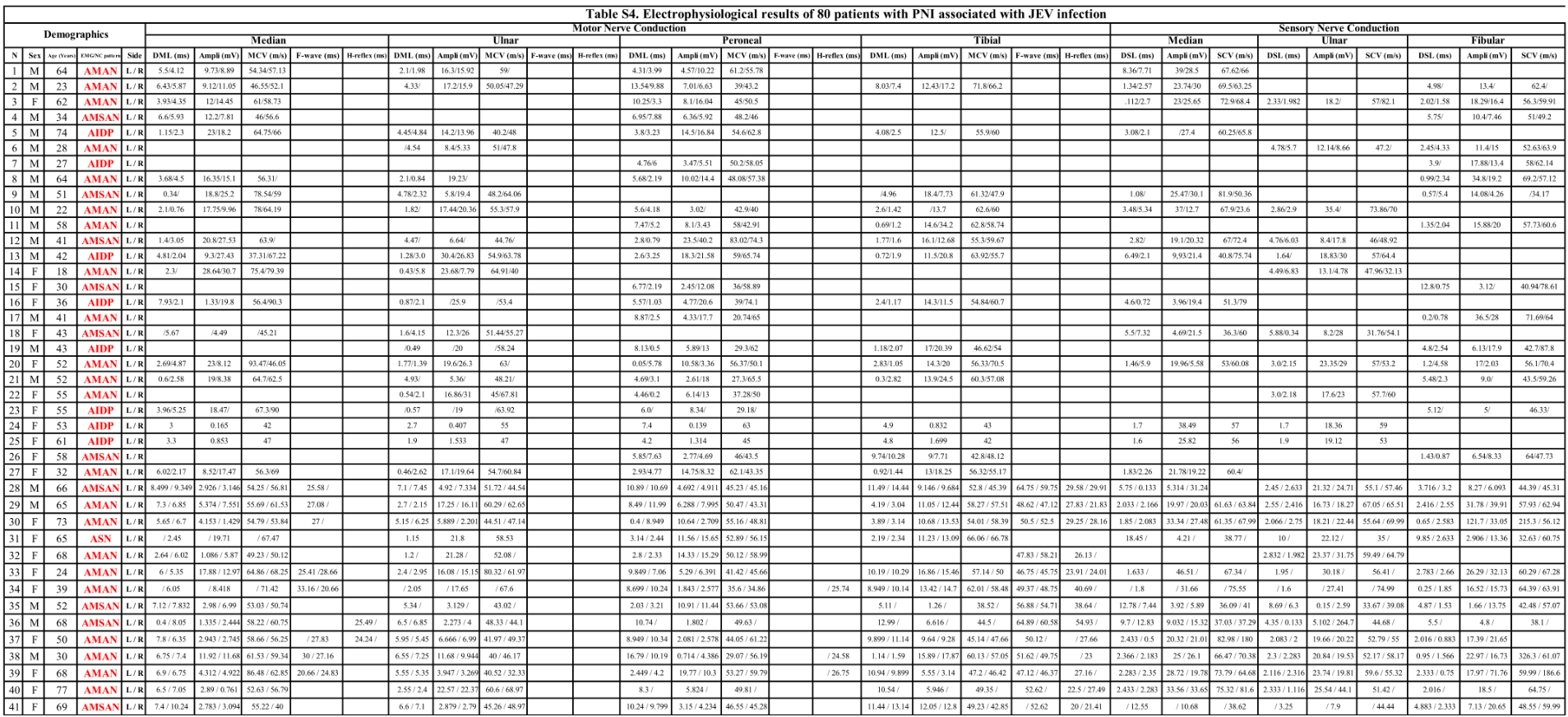


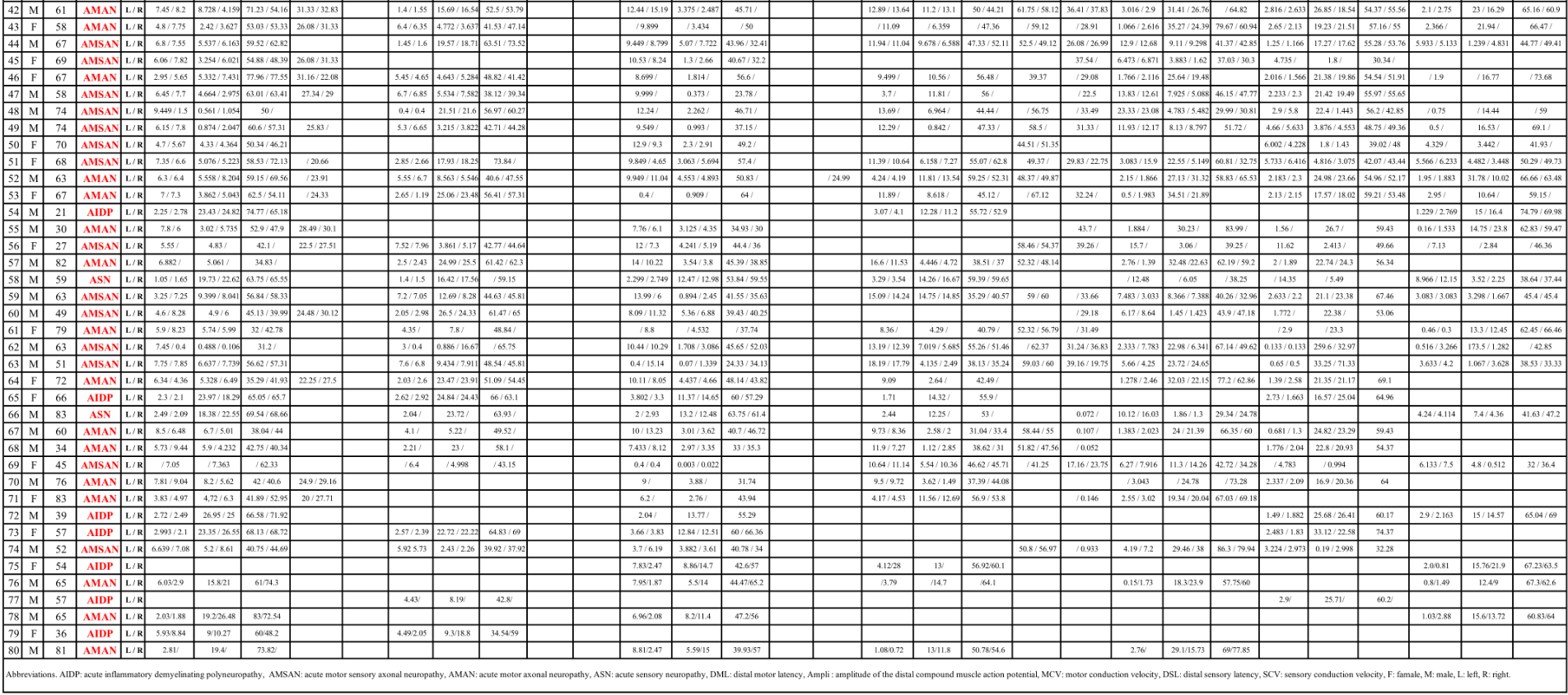


| Table S5. Motor and sensory nerve conduction outcomes in 80 patients with PNI associated with JEV infection. | | | | | | | | |
| --- | --- | --- | --- | --- | --- | --- | --- | --- |
| Motor Nerve Conduction Sensory Nerve Conduction  Parameters Be hospitalized Be hospitalized After discharge Parameters Be hospitalized Be hospitalized After discharge  (n_1_ = 56–73) (n_2_ = 70) (n_α_ = 17) (n_3_ = 52–56) (n_4_ = 27) (n_β_ = 17) | | | | | | | | |
| Median | DML (ms) N<3.3 | 6.3 | 7.0 | 4.0 | DSL (ms) N<3.5 | 5.5 | 9.6 | 4.0 |
|  | Ampli (mV) N>17 | 8.2 | 5.9 | 11.5 | Ampli (mV) N>19 | 18.1 | 11.4 | 22.9 |
|  | MCV (m/s) N>62 | 57 | 53 | 62 | SCV (m/s) N>58 | 56 | 44 | 67 |
| Ulnar | DML (ms) N<3.0 | 4.4 | 4.8 | 2.8 | DSL (ms) N<3.1 | 3.0 | 3.9 | 2.5 |
|  | Ampli (mV) N>15 | 11.5 | 10.2 | 15.2 | Ampli (mV) N>16 | 25.0 | 27.8 | 28.1 |
|  | MCV (m/s) N>50 | 47 | 45 | 58 | SCV (m/s) N>51 | 52 | 50 | 59 |
| Peroneal | DML (ms) N<4.1 | 8.0 | 9.2 | 5.7 | DSL (ms) N<3.0 | 3.7 | 5.1 | 2.5 |
|  | Ampli (mV) N>10 | 6.1 | 4.9 | 9.3 | Ampli (mV) N>12 | 18.0 | 10.6 | 30.7 |
|  | MCV (m/s) N>51 | 45 | 41 | 50 | SCV (m/s) N>57 | 66 | 47 | 76 |
| Tibial | DML (ms) N<4.3 | 8.8 | 9.6 | 5.6 |  |  |  |  |
|  | Ampli (mV) N>11 | 9.4 | 9.0 | 12.3 |  |  |  |  |
|  | MCV (m/s) N>52 | 48 | 46 | 56 |  |  |  |  |
| Abbreviations: DML, distal motor latency; Ampli, amplitude of the distal compound muscle action potential; MCV, motor conduction velocity; SCV, sensory conduction velocity.  n_1_ indicates 56 to 73 cases of motor nerve conduction during hospitalized, including 70 of median, 62 of ulnar, 73 of peroneal and 56 of tibial nerve.  n_2_ indicates 70 cases of motor nerve conduction abnormalities.  n_3_ indicates 52 to 56 cases of sensory nerve conduction during hospitalized, including 55 of median, 56 of ulnar and 52 of peroneal nerve.  n_4_ indicates 21 cases of sensory nerve conduction abnormalities.  n_α_ and n_β_ indicate both 17 cases of motor and sensory nerve conduction of 8 months after discharge. | | | | | | | | |

| Table S6. Neurological features of GBS patients associated with JEV infection. |
| --- |
| No. (%)  GBS  Children Adults  (n = 4) (n = 105) |
| Age, mean (SD, range), y 15 (9‒18) 57 (19‒83) |
| Male sex 1 (25) 58 (55) |
| Fever 4 (100) 104 (99) |
| Limb weakness followed by fever 1/1 (100) 41/74 (66) |
| Disturbance of consciousness 3 (75) 84 (80) |
| Median time from onset to disturbance of consciousness, (IQR),d 3 (1–6) 5 (2–8) |
| Trouble breathing 2 (50) 57 (54) |
| Median time from onset to trouble breathing, (IQR), d 2 (1–4) 3 (1–5) |
| Facial palsy |
| Unilateral facial palsy 0 18/26 (69) |
| Bilateral facial palsy 0 3/8 (38) |
| Blood pressure and heart rate decreased 0 4/6 (67) |
| Flaccid paralysis^a^ 4 (100) 105 (100) |
| Weakness of the respiratory muscle 3 (75) 93 (89) |
| Median time from JEV infection to onset of GBS symptoms, (IQR), days^b^ 6 (1–7) 4 (1–5) |
| Brain MRI |
| Unilateral or bilateral thalamus 3/4 (75) 79/99 (80) |
| Unilateral or bilateral cerebral peduncle 0 33/99 (33) |
| Unilateral or bilateral basal ganglia 0 24/99 (24) |
| Corpus callosum splenium 0 11/19 (58) |
| Unilateral or bilateral hippocampus 0 10/14 (71) |
| CSF albuminocytologic dissociation 3/4 (75) 74/102 (73) |
| Basis for neurologic diagnosis of Guillain–Barré syndrome^c^ |
| Brighton criteria level 1 3 (75) 79 (75) |
| Brighton criteria level 2 1 (25) 20 (19) |
| Brighton criteria level 3 0 6 (6) |
| Abnormal EMG 2 (50) 78 (74) |
| Simple MCV decreased 2 (100) 51 (65) |
| Simple SCV decreased 0 3 (4) |
| Both MCV and SCV decreased 0 24 (31) |
| Decreased CMAP amplitude 0 50 (64) |
| Disappeared F-waves or H-reflections 0 28 (36) |
| Diagnosis based on nerve-conduction studies and EMG^d^ 4 (100) 105 (100) |
| AIDP 2 (50) 10 (10) |
| AMAN 2 (50) 68 (65) |
| AMSAN 0 24 (23) |
| ASN 0 3 (3) |
| Antiglycolipid IgM detection |
| In serum 1 (25) 41 (39) |
| Antiglycolipid IgM–positive 1/1 (100) 12/41 (29) |
| Anti-GM1 IgM–positive 1/1 (100) 7/41 (17) |
| Anti-GM2 IgM–positive 1/1 (100) 6/41 (15) |
| Anti-GD1a IgM–positive 0 3/41 (7) |
| Anti-GD1b IgM–positive 0 3/41 (7) |
| In CSF 0 29 (28) |
| Anti-GM1 IgM–positive 0 1/29 (3) |
| Treatment |
| Intravenous immune globulin 2/4 (50) 53/92 (58) |
| ICU admission 3/4 (75) 91/102 (89) |
| Mechanical ventilation 3/4 (75) 82/95 (86) |
| Mean length of stay, (range), d |
| In hospital 17 (5–34) 29 (1–135) |
| In ICU 14 (4–30) 18 (1–85) |
| Median duration of mechanical ventilation, (IQR), d 12 (8–15) 17 (13–29) |
| Percentage of hospitalization time spent in ICU 88.2 66.4 |
| Outcome |
| Discharged alive 3 (75) 88 (84) |
| Died 1 (25) 17 (16) |
| Follow-up |
| Limb muscle weakness 3 (75) 61(58) |
| Limb muscle atrophy 1 (25) 16 (15) |
| Abbreviations: GBS, Guillain–Barré syndrome; JEV, Japanese encephalitis virus; AIDP, acute inflammatory demyelinating polyneuropathy; AMAN, acute motor axonal neuropathy; AMSAN, acute motor–sensory axonal neuropathy; IQR, interquartile range; MRI, magnetic resonance imaging; CSF, cerebrospinal fluid; EMG, electromyography; MCV, motor conduction velocities; SCV, sensory conduction velocities; CMAP, compound muscle action potential; ASN, acute sensory neuropathy; ICU, intensive care unit. Percentages may not total 100 because of rounding.  ^a^ Flaccid paralysis is the main clinical feature of peripheral nerve injury. It is characterized by decreased muscle strength, hypomyotonia, areflexia or decreased reflexes, and negative pyramidal signs.  ^b^ The onset of symptoms of the GBS was defined as the first day of onset of limb weakness, sensory symptoms, facial paralysis, or other neurologic symptoms.  ^c^ Brighton criteria levels indicate the certainty of a diagnosis of the GBS. A level 1 diagnosis is supported by nerve-conduction studies and the presence of albuminocytologic dissociation in the CSF. A level 2 diagnosis is supported by either a CSF white-cell count of less than 50 cells per cubic millimeter (with or without an elevated protein level) or by results of nerve-conduction studies that are consistent with the GBS, if the CSF white-cell count is unavailable). A level 3 diagnosis is based on clinical features without support from nerve-conduction or CSF studies.  ^d^ In 20 of the 109 patients with GBS, the EMG diagnostic outcomes was complete, despite the lack of detailed parametric data. The subtype of GBS can be determined based on the EMG results and clinical characteristics of the patients. |

**Supplementary figures**

**
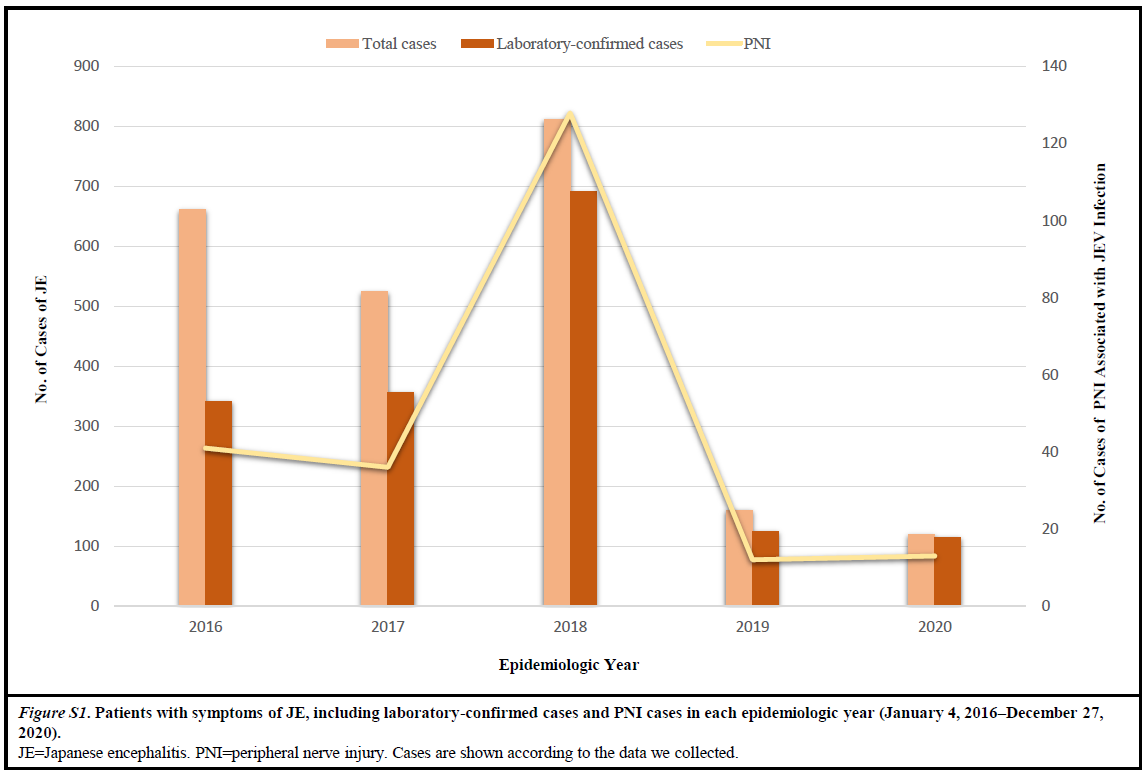
**

**
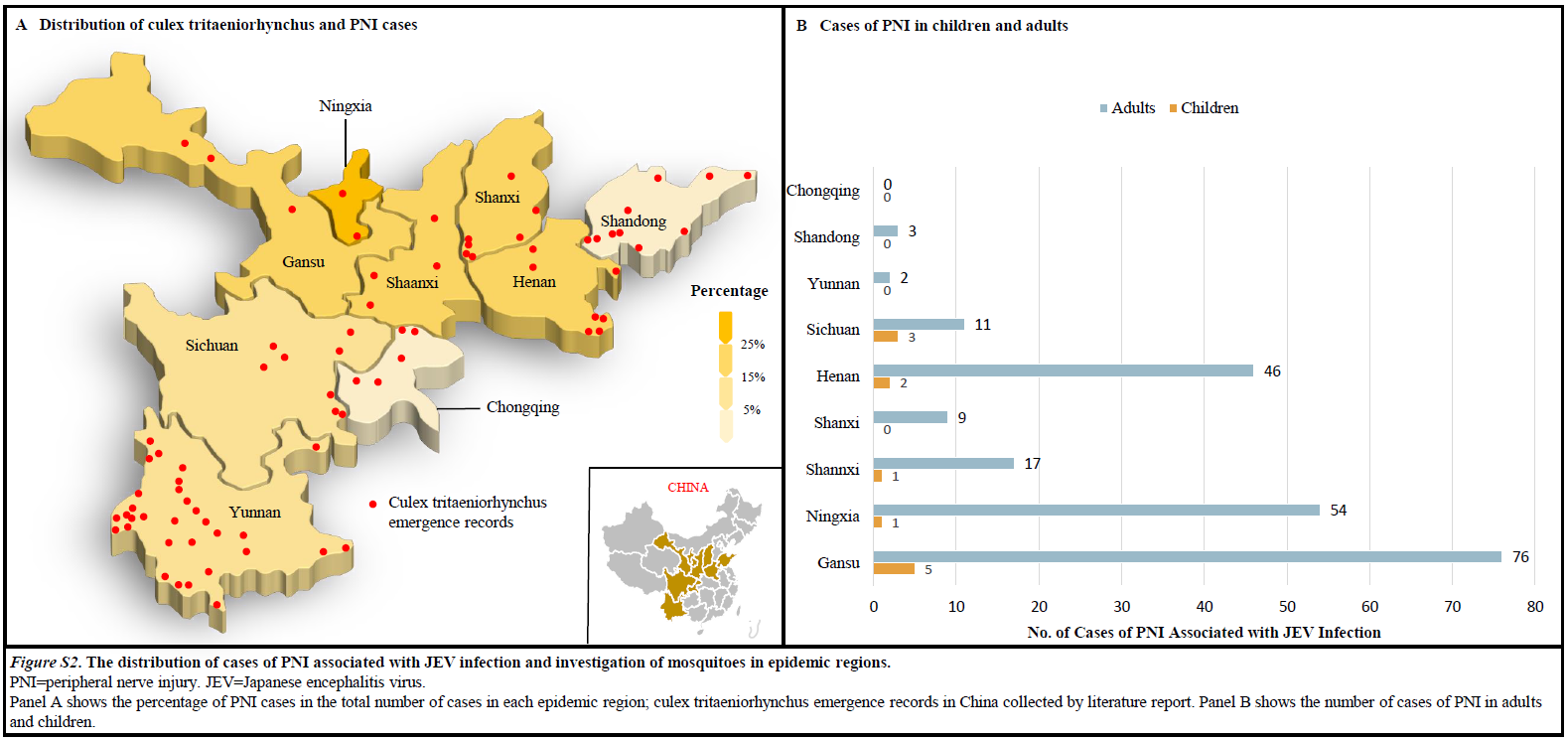
**

**
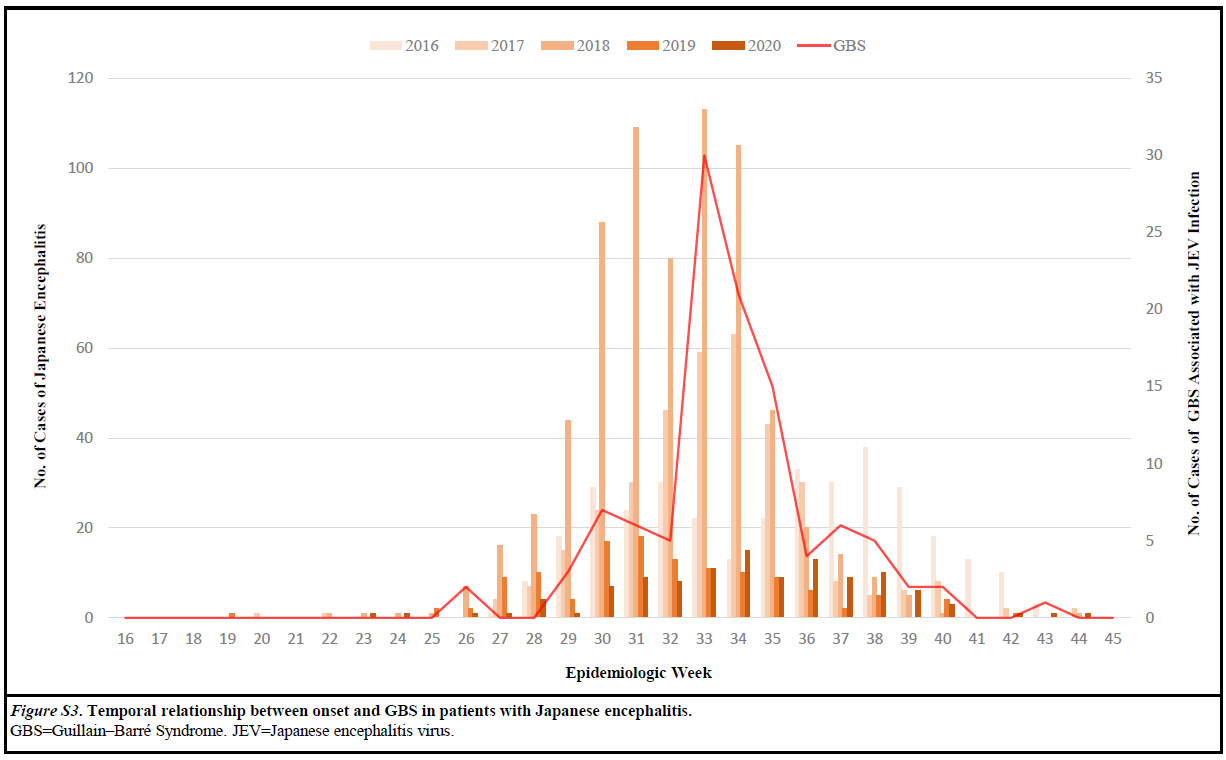
**


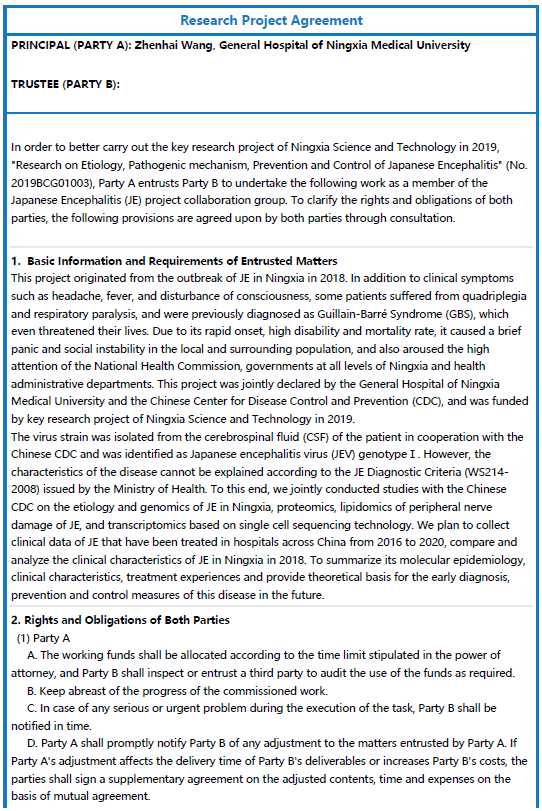


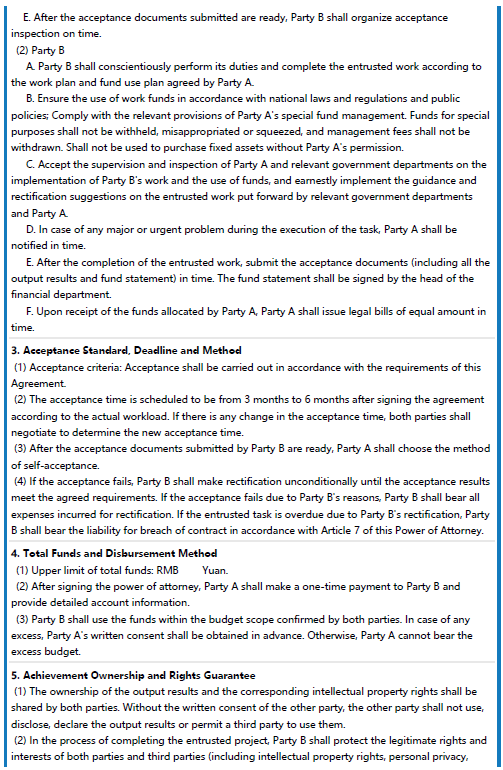


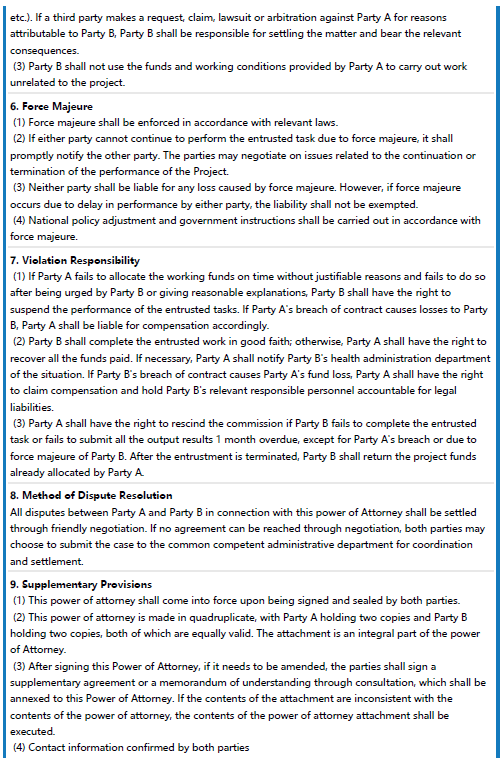


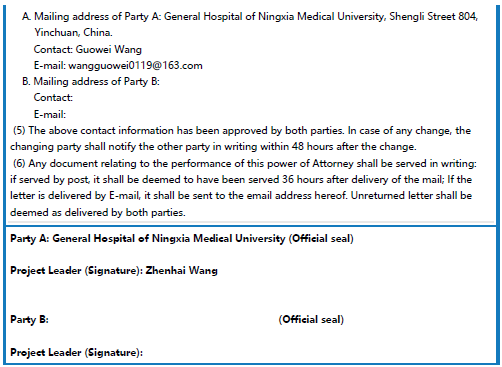


**Figure S4.** **The research project agreement of this study.**

The General Hospital of Ningxia Medical University, as the entrusted unit, signed cooperation agreements with other members.

**Form**


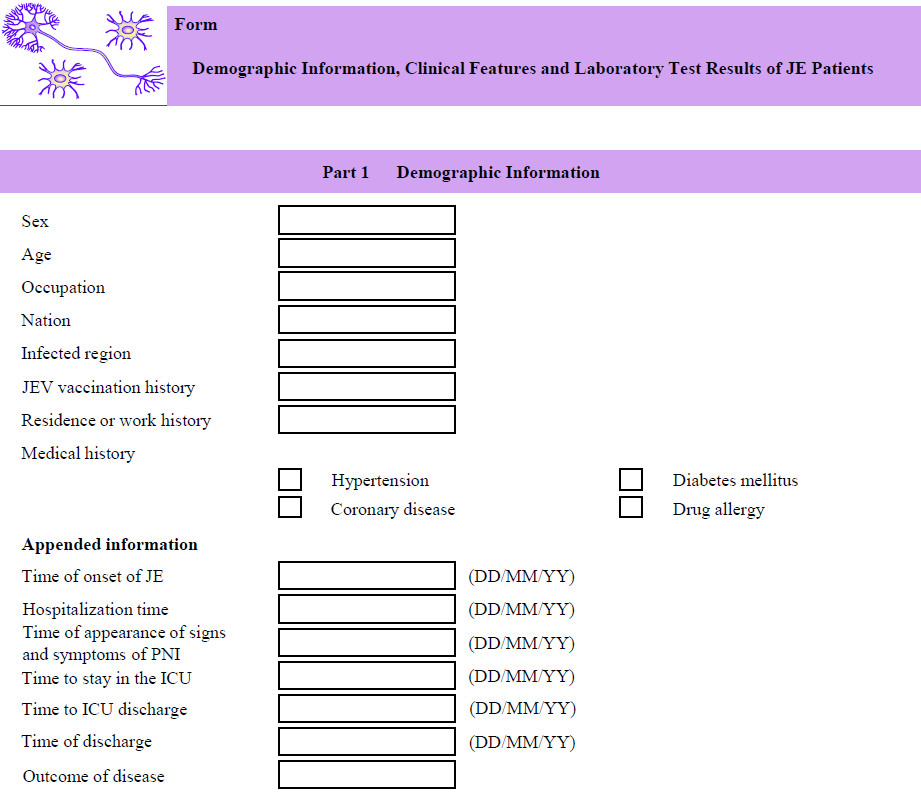


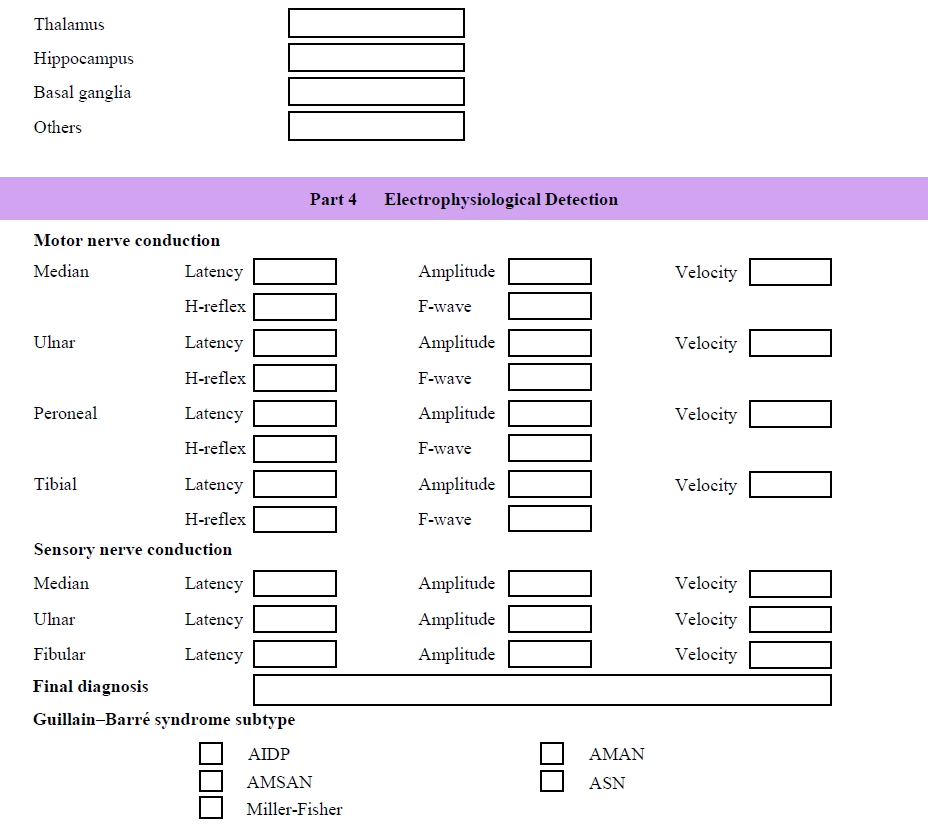


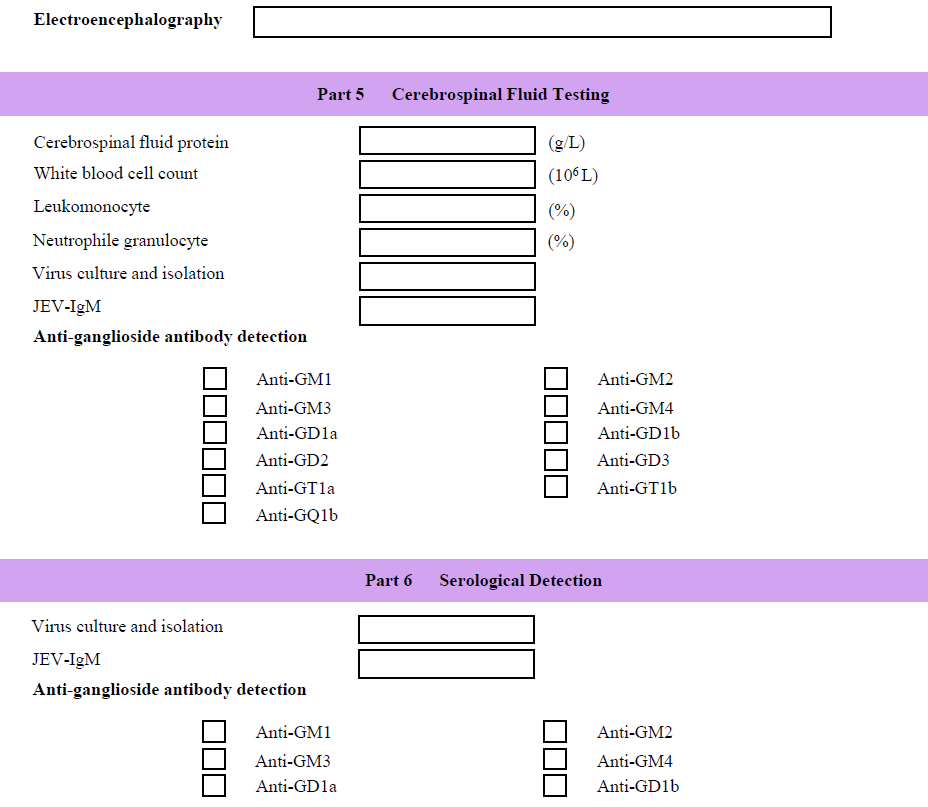


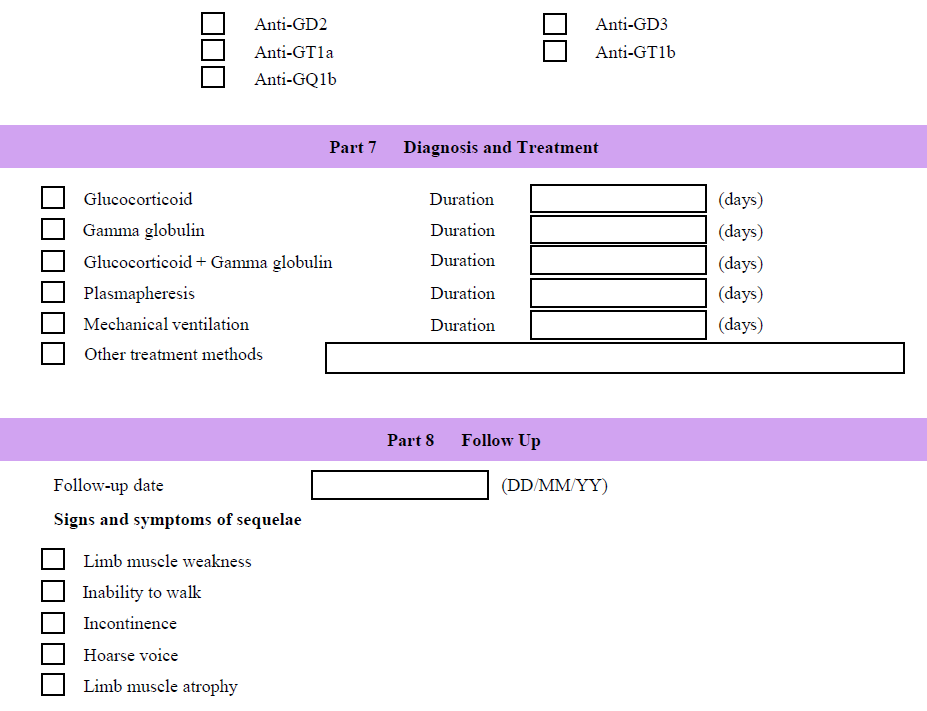


**Reference**

1 Qiuling Zang, Yating Wang, Junshuang Guo, et al. Treatment of Severe Japanese Encephalitis Complicated With Hashimoto's Thyroiditis and Guillain-Barré Syndrome With Protein A Immunoadsorption: A Case Report. Front Immunol **2022**; 12: 807937.

2 Wang Miao, Junshuang Guo, Shuyu Zhang, et al. The Effect of a Combined Ganciclovir, Methylprednisolone, and Immunoglobulin Regimen on Survival and Functional Outcomes in Patients With Japanese Encephalitis. Front Neurol **2021**; 12: 711674.
